# SIGHT—A System for Solvent‐Tight Incubation and Growth Monitoring in High Throughput

**DOI:** 10.1002/elsc.202400037

**Published:** 2024-09-11

**Authors:** Jakob Rönitz, Felix Herrmann, Benedikt Wynands, Tino Polen, Nick Wierckx

**Affiliations:** ^1^ Institute of Bio‐ and Geosciences IBG‐1: Biotechnology Forschungszentrum Jülich Jülich Germany

**Keywords:** Growth Profiler, high throughput cultivation, *Pseudomonas taiwanensis*, solvent tolerance, styrene

## Abstract

Physiological characterization of microorganisms in the context of solvent tolerance is a tedious process with a high investment of manual labor while often being limited in throughput capability simultaneously. Therefore, we developed a small‐scale solvent‐impervious cultivation system consisting of screw cap‐sealed glass vials in combination with a 3D‐printed vial holder for the Growth Profiler (EnzyScreen) platform. Components and cultivation conditions were empirically tested, and a suitable setup was found for the intended application. To demonstrate the capability of this cultivation system, an adaptive laboratory evolution was performed to further increase the tolerance of *Pseudomonas taiwanensis* GRC3 toward styrene. This approach yielded heterogenic cultures with improved growth performances in the presence of styrene from which individual clones were isolated and characterized in high throughput. Several clones with improved growth in the presence of 1% (v/v) styrene were analyzed through whole‐genome sequencing, revealing mutations in the co‐chaperone‐encoding gene *dnaJ*, RNA polymerase α subunit‐encoding gene *rpoA*, and loss‐of‐function mutations in the *ttgGHI* solvent efflux pump repressor encoded by *ttgV*. The developed cultivation system has proven to be a very useful extension of the Growth Profiler, as it reduces manual workload and allows high‐throughput characterization.

**Abbreviations**
ALE: adaptive laboratory evolution
CTD: C‐terminal domain
DIY: do it yourself
LB: lysogeny broth
MSM: mineral salt medium
OTR: oxygen transfer rate
PTFE: polytetrafluoroethylene
RNA: ribonucleic acid
RNAP: ribonucleic acid polymerase
RND: resistance‐nodulation‐division
SIGHT: solvent‐tight incubation and growth monitoring in high throughput

## 1 Introduction

Increasing sustainability of the chemical industry in the scope of “Green Chemistry” is a major challenge but provides an opportunity to reduce the environmental footprint of this sector and address global issues like climate change. Biotechnological production of chemicals from renewable feedstocks is an important part of this concept, as conventional synthesis is mostly based on fossil resources [1]. However, bio‐based production is currently only possible for a limited number of platform chemicals, and major building blocks such as ethylene, propylene, butadiene, and the aromatics are still inaccessible [2]. Among the latter group, approximately 30 million tons of styrene were globally produced in 2021, with an expected growth of the market to 50 million tons by 2030 [3]. Styrene is mostly produced by dehydrogenation of fossil ethylbenzene [4] and used for various polymers, such as polystyrene (PS), styrene acrylonitrile (SAN), acrylonitrile butadiene styrene (ABS), and styrene‐butadiene rubber (SBR). However, due to the solvent characteristics of styrene and its associated high toxicity, microbial production processes are highly challenging [5, 6, 7]. Like many other aromatic compounds, styrene can easily enter cell membranes upon contact with the cell surface [8]. The accumulation of solvents in the cell membrane causes increased fluidity that can lead to its disruption, resulting in the loss of the proton gradient and eventually cell death [9]. An important parameter for the diffusion of a compound into cell membranes is its logP_O/W_ value, which is the logarithmic partition coefficient in a system containing *n*‐octanol and water. In general, compounds within the logP_O/W_ range of 1.5–4 are considered highly toxic for most microorganisms [10], as they are still water soluble to some extent. Therefore, a robust host organism with high solvent tolerance is required to overcome product toxicity limitations in the context of biosynthesis of styrene, featuring a logP_O/W_ of 3.05 [11].

Some bacteria of the *Pseudomonas* clade have a high natural tolerance toward solvents and therefore display suitable candidate hosts to establish styrene bioproduction chassis. Especially the *Pseudomonas putida* strains DOT‐T1E and S12 have been intensively studied regarding their solvent tolerance in the past [10]. These strains feature a variety of intrinsic mechanisms to counteract solvent toxicity such as modulation of the fatty acid composition in the cell membrane by cis‐trans‐isomerases [12, 13], back folding of damaged proteins by chaperones [14] and active extrusion via efflux pumps [9, 15]. Efflux pumps of the resistance–nodulation–division (RND) family represent the most effective solvent detoxification mechanism in Pseudomonads and consist of three components—an inner membrane protein, an outer membrane channel, and a periplasmic adaptor protein [9]. Extrusion of solvents via RND‐type efflux pumps is driven by the proton gradient and hence energy dependent [16]. To compensate for this increased energy demand, some Pseudomonads are able to upregulate their energy metabolism under stress conditions by increasing glucose uptake and NADH oxidation rates to sustain the proton motive force [17]. *Pseudomonas taiwanensis* VLB120 is a robust strain that has been used for epoxidation of styrene [18, 19] as well as for production of a wide range of other aromatics [20, 21] in the past, proving its suitability for applications involving solvents. Based on this strain, a set of three genome‐reduced chassis strains with different levels of solvent tolerance was previously constructed [22]. Out of these three strains, *P. taiwanensis* GRC3 possesses the *ttgGHI* genes encoding a solvent efflux pump with the corresponding regulatory *ttgVW* operon for inducible solvent tolerance.

Evaluation of these tolerance mechanisms is not trivial due to the volatility of many solvents, which requires the use of closed cultivation systems to prevent evaporation over time. Additionally, all parts of the system that come into direct contact with the solvent should consist of inert materials such as glass and polytetrafluoroethylene (PTFE). Polymers like polypropylene (PP), polyethylene (PE), or polystyrene (PS) often used for high‐throughput cultivation are not suitable as most solvents are absorbed by these materials and potentially dissolve or damage them over time. Hence, the handling of the cultivation system during experiments is not trivial. Established systems such as closed shake flasks or Boston bottles with Mininert valves require manual sampling with a syringe, making optical density (OD_600_) measurements for the characterization of cell growth very labor‐intensive and dangerous, limiting the number of strains and replicates that can be handled in parallel.

Here, we developed and tested a DIY cultivation system that meets the requirements to contain volatile compounds and allows studying solvent tolerance of microorganisms in high throughput. The system was named solvent‐tight incubation and growth monitoring in high throughput (SIGHT) and is compatible with the Growth Profiler (EnzyScreen) screening platform. To evaluate the SIGHT system, we performed adaptive laboratory evolution (ALE) of *P. taiwanensis* GRC3 toward increased styrene tolerance. Isolated clones with improved styrene tolerance were then analyzed through whole‐genome sequencing to identify potential new targets for reverse engineering approaches.

## 2 Material and Methods

### 2.1 Bacterial Strains, Media, and Cultivation Conditions

All used *P. taiwanensis* strains (Table 1) were routinely streaked on lysogeny broth (LB) agar containing 10 g/L tryptone, 5 g/L yeast extract, 5 g/L NaCl, and 15 g/L agar‐agar (Carl Roth GmbH + Co. KG, Germany) and were incubated overnight at 30°C prior to the inoculation of liquid cultures. Growth and tolerance experiments were performed using mineral salt medium (MSM), modified based on Hartmans et al. [23], containing 3.88 g/L K_2_HPO_4_, 1.63 g/L NaH_2_PO_4_, 2 g/L (NH_4_)_2_SO_4_, 10 mg/L EDTA, 100 mg/L MgCl_2_ ⋅ 6H_2_O, 2 mg/L ZnSO_4_ ⋅ 7H_2_O, 1 mg/L CaCl_2_ ⋅ 2H_2_O, 5 mg/L FeSO_4_ ⋅ 7H_2_O, 0.2 mg/L Na_2_MoO_4_ ⋅ 2H_2_O, 0.2 mg/L CuSO_4_ ⋅ 5H_2_O, 0.4 mg/L CoCl_2_ ⋅ 6H_2_O, 1 mg/L MnCl_2_ ⋅ 2H_2_O, and 20 mM glucose. For tolerance experiments, styrene (≥99%, Sigma‐Aldrich, USA) was added in indicated amounts.

**TABLE 1:** Bacterial strains used in this study. Catalogization numbers are indicated by MiCat #.

Strain name	Genotype	Characteristic	Reference
*P. taiwanensis* GRC2	∆pSTY ∆prophage1/2::*ttgGHI*, ∆prophage3, ∆prophage4, ∆flag1, ∆flag2, ∆lap1, ∆lap2, ∆lap3		Wynands et al. [22], MiCat #4
*P. taiwanensis* GRC3	∆pSTY ∆prophage1/2::*ttgVWGHI*, ∆prophage3, ∆prophage4, ∆flag1, ∆flag2, ∆lap1, ∆lap2, ∆lap3		Wynands et al. [22], MiCat #5
*P. taiwanensis* GRC3 ALE I 1 mM clone 1	∆pSTY ∆prophage1/2::*ttgVWGHI*, ∆prophage3, ∆prophage4, ∆flag1, ∆flag2, ∆lap1, ∆lap2, ∆lap3, *dnaJ*951_952insG	Frameshift in *dnaJ* leading to early stop	This study, MiCat #2714
*P. taiwanensis* GRC3 ALE I 1.5 mM clone 1	∆pSTY ∆prophage1/2::*ttgVWGHI*, ∆prophage3, ∆prophage4, ∆flag1, ∆flag2, ∆lap1, ∆lap2, ∆lap3, *dnaJ*951_952insG	Frameshift in *dnaJ* leading to early stop	This study, MiCat #2715
*P. taiwanensis* GRC3 ALE I 1.5 mM clone 2	∆pSTY ∆prophage1/2::*ttgVWGHI*, ∆prophage3, ∆prophage4, ∆flag1, ∆flag2, ∆lap1, ∆lap2, ∆lap3, *ttgV*682_687delGAGC	Frameshift in *ttgV* leading to early stop	This study, MiCat #2716
*P. taiwanensis* GRC3 ALE I 1.5 mM clone 4	∆pSTY ∆prophage1/2::*ttgVWGHI*, ∆prophage3, ∆prophage4, ∆flag1, ∆flag2, ∆lap1, ∆lap2, ∆lap3, *dnaJ*951_952insG	Frameshift in *dnaJ* leading to early stop	This study, MiCat #2717
*P. taiwanensis* GRC3 ALE I 1% (v/v) clone 3	∆pSTY ∆prophage1/2::*ttgVWGHI*, ∆prophage3, ∆prophage4, ∆flag1, ∆flag2, ∆lap1, ∆lap2, ∆lap3, *rpoA^D257H^*, *ttgV*682_687delGAGC	D257H in *rpoA*, frameshift in ttgV leading to early stop	This study, MiCat #2718
*P. taiwanensis* GRC3 ALE III 1.5 mM clone 1	∆pSTY ∆prophage1/2::*ttgVWGHI*, ∆prophage3, ∆prophage4, ∆flag1, ∆flag2, ∆lap1, ∆lap2, ∆lap3, *ttgV*39_43delins[NC_022738:g.5597919_5623638]	Tn insertion (25.7 kb) in *ttgV* → loss of function, gene duplications (e.g., putative heat shock proteins)	This study, MiCat #2719

*P. taiwanensis* pre‐cultures were inoculated from LB agar plates and cultivated in square 24‐well System Duetz plates (EnzyScreen, the Netherlands) using 1 mL MSM. Plates were then incubated at 30°C and 300 rpm overnight. Main cultures were inoculated to an initial OD_600_ of 0.05 using overnight cultures and incubated in closed glass vials using the SIGHT system and the Growth Profiler 960 (EnzyScreen, the Netherlands) set to 30°C and 225 rpm. Culture volumes using the prototype SIGHT system with 6 mL vials are indicated for each experiment. For the finalized SIGHT system, 5 mL vials were used with 600 µL liquid volume unless stated otherwise.

### 2.2 Components of the SIGHT System

The developed SIGHT system for the Growth Profiler 960 (EnzyScreen, the Netherlands) consists of a 3D‐printed vial holder with 6 × 4 layout and closed glass vials. Online growth monitoring is possible due to cutouts in the bottom of the vial holder, enabling image analysis and calculation of growth curves based on Green Values by the Growth Profiler. To obtain a high Green Value range, a white PETG filament was used to print the vial holder. Sterilization of the glass vials was achieved through autoclaving at 121°C for 20 min.

The prototype of this cultivation system was designed for glass vials of 6 mL total volume. These vials were made from commercially available glass vials (vial G8 clear, 15‐425 thread, 8 mL, diameter: 17 mm, height: 61 mm, Art. No. 300130, CS Chromatographie Service GmbH, Germany), which were shortened at the bottom part of the cylinder and fused to a glass plate of 1.75 mm thickness by a glass blower. The shortened flat bottom vials were used in combination with screw caps and PTFE septum (screw cap G15 + DS G15, Art. No. 300330, CS Chromatographie Service GmbH, Germany) to obtain a closed system.

The final version of the vial holder was designed with holes of 15 mm diameter to fit commercially available glass vials (“sample vials ROTILABO, ND13 thread, 4 mL, diameter: 14.7 mm, height: 45 mm,” Art. No. LC31.1, Carl Roth GmbH + Co. KG, Germany), which provide a closed and solvent proof system in combination with suitable screw caps and a PTFE‐coated septum (screw caps ROTILABO, ND13 thread, septum: butyl (red)/PTFE (gray), Art. No. TY89.1, Carl Roth GmbH + Co. KG, Germany). The total volume of this system was determined to be 4.937 (±0.058) mL (referred to as 5 mL vials) by filling the vials with water (*n* = 10), determining the mass of water using a LA310S fine balance (Sartorius AG, Germany) and calculating the corresponding volume considering a water density of 0.9980 g cm^3−1^ at 21°C. The STL file of the final version of the vial holder is available at https://www.thingiverse.com/thing:5416902.

### 2.3 Generation of Green Value–Based Growth Curves

Green Value–based growth curves were obtained using the Growth Profiler 960 (EnzyScreen, the Netherlands) and the corresponding image analysis software GP960Viewer (version 1.1.1.0). The device takes photos of the transparent bottom of incubated well plates—or transparent glass vials in the case of this study. The software analyzes the number of green pixels in a defined area of these photos, which correlates with OD_600_ of the culture and allows growth monitoring.

### 2.4 Calculation of Styrene Concentration in a Medium

Styrene concentrations in the medium were calculated by applying Henry's law and assuming a closed system in an equilibrium state. A partition coefficient describing the ratio between styrene concentration in the headspace and aqueous phase (*k_air/medium_* = 0.148 at 30°C) [24] and a solubility limit of 2.8 mM [25] were used. Further addition of styrene to the system will lead to the formation of a second organic phase on top of the aqueous phase. Equation (1) describes the distribution of styrene between the aqueous and gas phases within a closed system [26] and was rearranged resulting in Equation (2). Equation (2) was then used to calculate the required volume of styrene to be added to the system to achieve the desired concentration in the aqueous phase when the equilibrium state is reached.

(1) $$c_{a}\times \;V_{m}=n_{m+a}\times \;\frac{k_{air/medium}}{k_{air/medium}\;\times \;\left(\frac{V_{a}}{V_{m}}\right)\;+\;1},\;\mathrm{and}$$

(2) $$V_{sty}=V_{m}\times \left(k_{\frac{air}{medium}}\times \left(\frac{V_{a}}{V_{m}}\right)+1\right)\times c_{m}\times M_{sty}\times \frac{1}{\rho_{sty}},$$

where *c_a_ = *styrene concentration in air (mol × L^−1^), *c_m_* = styrene concentration in medium (mol × L^−1^), *n_m+a_* = styrene in whole system (mol), *n_m_* = styrene in medium (mol), *n_a_* = styrene in air (mol), *V_m_* = volume medium (L), *V_a_* = volume air (L), *M_sty_* = molar mass styrene (g × mol^−1^), *ρ_sty_* = density styrene (g × (cm^3^)^−1^), *k_air/medium_* = partition coefficient at 30°C (L_*medium*_ × L_*air*_^−1^), and *V_sty_* = volume styrene in system (mL).

### 2.5 Analytical Methods

The OD_600_ of bacterial cultures was measured at *ʎ* = 600 nm using an Ultrospec 10‐cell density meter (Biochrom Ltd., UK).

Glucose and gluconate concentrations in culture supernatant were quantified by high‐performance liquid chromatography (HPLC) using an Agilent 1260 Infinity II equipped with a diode array detector (DAD) and refractive index detector (RID) (Agilent Technologies, Inc., USA). Cultures were centrifuged at 16,000 × *g* for 5 min and supernatant was collected for analysis. A Metab‐AAC column (length: 300 mm, inner diameter: 7.8 mm) equipped with Metab‐AAC pre‐column (length: 40 mm; inner diameter: 7.8 mm) (ISERA GmbH, Germany) was used and 5 mM H_2_SO_4_ was applied as mobile phase at a constant flow rate of 0.6 mL min^−1^ at 40°C. Gluconate was quantified using the DAD signal at 210 nm and glucose using the RID signal. Both compounds co‐elute with this method; however, only gluconate gives a signal at 210 nm in the DAD. Therefore, glucose concentration was calculated by subtracting the gluconate concentration from the mixed signal (glucose and gluconate) obtained from the RID.

### 2.6 Whole‐Genome Sequencing and Data Analysis

For DNA isolation, single clones were grown in an LB medium. gDNA was isolated with the Monarch Genomic DNA Purification Kit (New England Biolabs, USA). The resulting gDNA concentration was determined via a Qubit 2.0 fluorometer (Thermo Fisher Scientific, USA). From the prepared gDNA, 1 µg was used for library preparation employing the NEBNext Ultra II DNA Library Prep Kit (New England Biolabs, USA). Via qPCR using the KAPA library quantification kit (Peqlab, Germany), the library was evaluated and then normalized via pooling. After in‐house sequencing (paired‐end) using MiSeq (Illumina, USA) with a read length of 2 × 150 bases, the demultiplexed FASTQ output files were processed with the CLC Genomic Workbench software (Qiagen, the Netherlands). For reads mapping and variants calling, a *P. taiwanensis* GRC3 reference genome was used, which was generated from a *P. taiwanensis* VLB120 genome (NCBI sequence reads archive, accession number: SRX6455847) by manual introduction of respective modifications as described by Wynands and co‐workers [22]. Mutations and deletions were assessed manually regarding their specific occurrence between the different samples and their relevance. Identified genomic mutations were verified by the PCR amplification of the respective locus using the Q5 High‐Fidelity DNA Polymerase (New England Biolabs, USA), purification of PCR products using the Monarch DNA Gel Extraction Kit (New England Biolabs, USA), and subsequent Sanger sequencing of the PCR products using Mix2Seq Kit NXP (Eurofins Genomics, Germany). Primers used for amplification and sequencing of PCR products are listed in Table S1.

## 3 Results

### 3.1 Cultivation System Development

**FIGURE 1:**
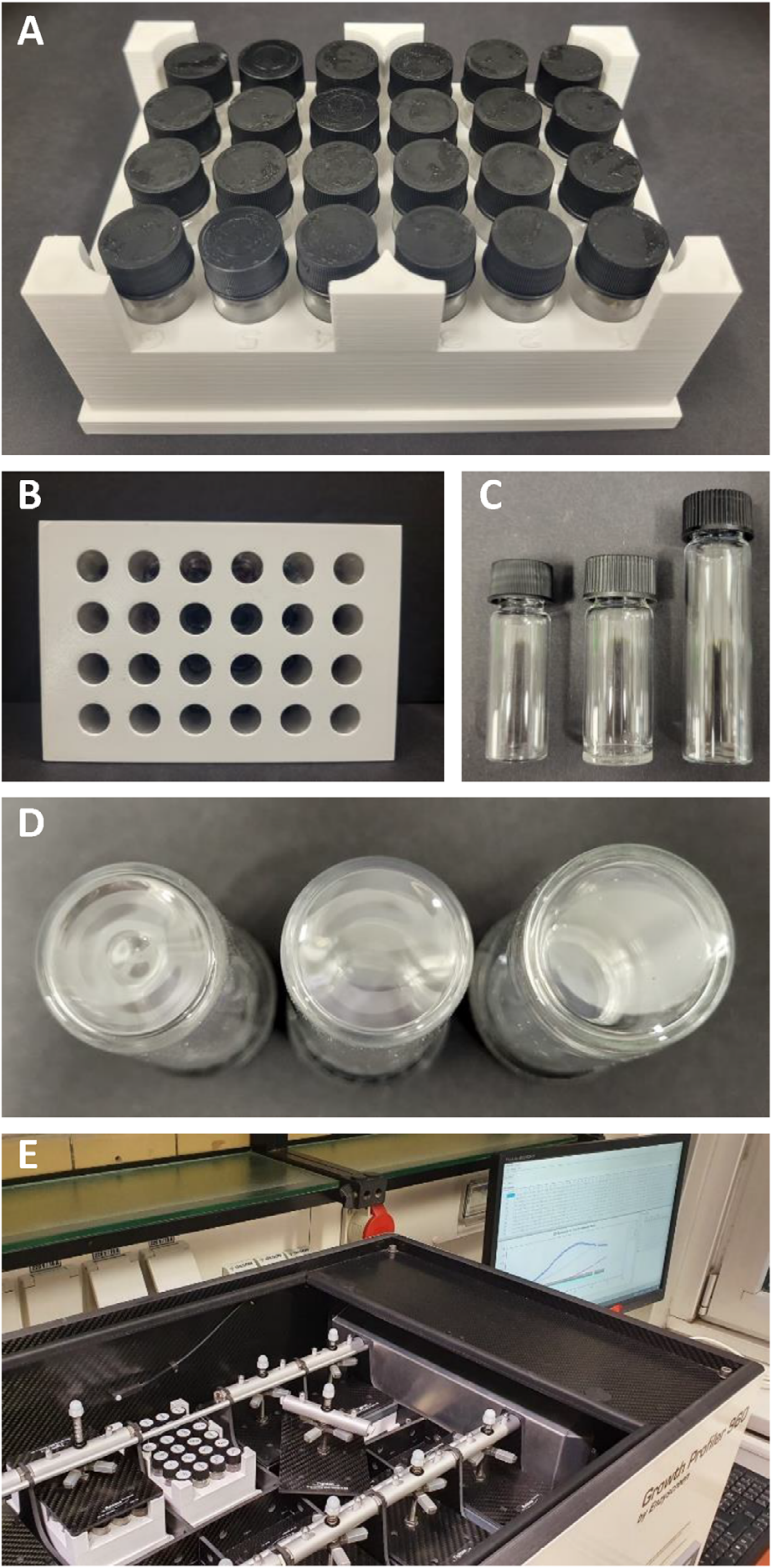
SIGHT cultivation system designed for the Growth Profiler. (A) Top view of the 3D‐printed holder with standard well plate footprint and capacity for 24 glass vials. (B) Bottom view of vial holder, cutouts allow image analysis and online growth monitoring using the Growth Profiler. (C) Glass vial types used during prototype development. Left: commercial 5 mL vial, center: custom‐made 6‐mL flat–bottom vial, right: commercial 8 mL glass vial used for crafting of 6 mL vials by shortening and fusion to flat glass plate. (D) Bottom shapes of different glass vial types. Left: 5 mL commercial vial drop shaped bottom, center: 5 mL commercial vial curved bottom, right: 6 mL custom‐made vial flat bottom. (E) Growth Profiler used in combination with solvent‐tight cultivation system.

**FIGURE 2:**
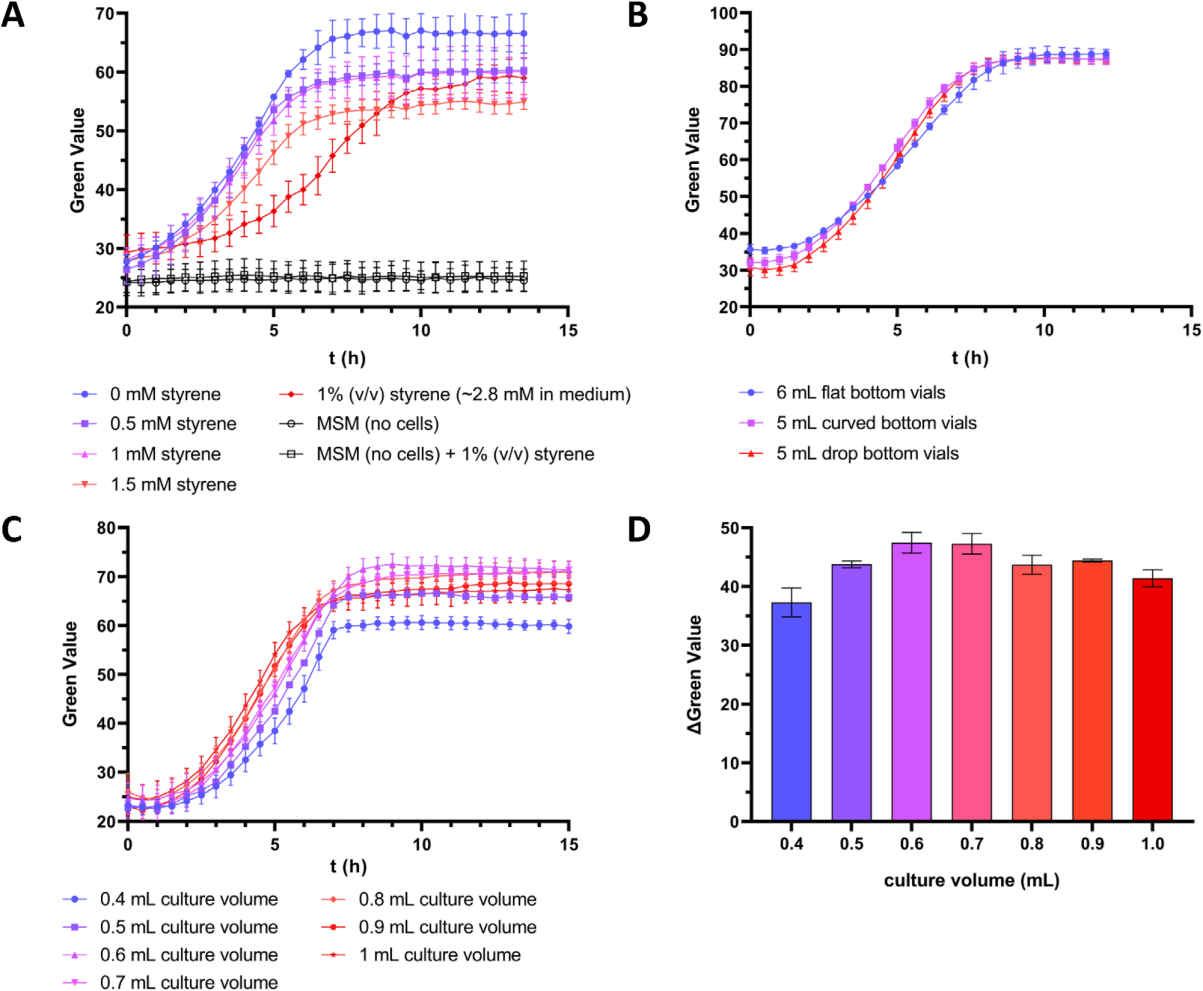
Growth of *P. taiwanensis* GRC2 in MSM using closed glass vials, initial OD_600_ was adjusted to 0.05 in all cultures. (A) Cell growth in the presence of different styrene concentrations using 6 mL flat‐bottom glass vials (hand‐crafted) with 1 mL liquid volume. (B) Testing different types of glass vials for the SIGHT system using MSM without addition of styrene. A culture volume of 1 mL was used for 6 mL glass vials, which was scaled down to 833 µL for the 5 mL vials to maintain a constant medium‐to‐headspace ratio. (C) Direct comparison of growth curves obtained from cultivation of *P. taiwanensis* GRC2 in 5 mL glass vials using different culture volumes without styrene addition. (D) Change in Green Value range (ΔGreen Value) between *t*  =  0 h and *t* = 10 h for different culture volumes. The data represents the mean of three biological replicates (*n* = 3). Error bars indicate the standard deviation of the mean.

The SIGHT cultivation system developed here consists of a 3D‐printed rack with a standard well plate footprint for 24 glass vials (Figure 1A). Cutouts on the bottom (Figure 1B) allow image analysis and online growth monitoring using the Growth Profiler (EnzyScreen) platform (Figure 1E). Growth curves based on Green Value are generated by analysis of green pixels within a defined area of captured photos, which correlates with biomass formation in the culture. The first working prototype of the cultivation system was designed for glass vials with a diameter of 17 mm and a total volume of 6 mL. These vials were hand‐crafted by a glass blower to obtain a flat bottom by shortening the tube of commercially available 8 mL vials and fusion of the bottom part to a flat glass plate (Figure 1C,D). This was done to maximize the accuracy of bottom–up image analysis, while still allowing the vials to fit the maximum height of plates for the Growth Profiler. This setup was tested through the cultivation of the constitutively solvent‐tolerant strain *P. taiwanensis* GRC2 (Δ*ttgVW*) using 1 mL MSM supplemented with 20 mM glucose as the sole carbon source (Figure 2A). Styrene was added in aqueous concentrations ranging from 0 mM up to saturation of the medium and formation of a second organic phase, which corresponds to 2.8 mM dissolved in the aqueous phase. To exclude the interference of the second phase of styrene with Green Values, control vials only containing MSM and MSM with the addition of 1% (v/v) of styrene were also monitored.

Based on the data obtained from using the prototype SIGHT cultivation system (Figure 2A), several conclusions were drawn: (i) The quality of the photos taken from the bottom of the vial holder is sufficient for image analysis by the Growth Profiler to produce growth curves based on Green Values. (ii) Styrene dose‐dependent growth inhibition of the cultures is detectable. (iii) The presence of styrene in the medium does not interfere with the measured Green Values (negative controls).

To make the SIGHT system more accessible to other labs, the prototype of the vial holder was adapted for the use of commercially available glass vials with a total volume of 5 mL. However, the bottoms of these commercial 5 mL vials are not flat, and a batch‐dependent variation of the bottom shape was observed (Figure 1D). Therefore, the influence of two different typical bottom shapes (“curved” and “drop”) on obtained Green Values was examined and compared to the 6 mL custom‐made benchmark vials. For this comparison, *P. taiwanensis* GRC2 was cultivated in all the three vial types in parallel (Figure 2B) using the same medium‐to‐headspace ratio to compensate for the difference in the total volume of the vials.

The obtained Green Value range was very comparable between the 6 mL custom‐made vials with flat bottoms and the 5 mL commercial vials. Additionally, the variation in the bottom shape of the commercial vials did not interfere with the obtained Green Values. Based on these results, commercial 5 mL glass vials were used subsequently. The robustness of the image analysis, regardless of the bottom shape, and the quality of resulting growth curves inspired the design of further prototype racks for different‐sized vials. These prototypes have capacity for 48 vials of 2 mL volume and 12 vials of 11 mL volume, respectively. Further information is available in Figures S2–S4 and Table S2 in the Supporting Information.

After finalizing the 24‐vial SIGHT system, the optimal medium‐to‐headspace ratio was empirically determined to find a balance between the aeration of the cultures and the Green Value range. The use of higher culture volumes correlates with lower headspace volumes and—because the vials are closed hermetically—absolute oxygen availability. It should also be noted that the oxygen transfer rate (OTR) between the gas phase and medium will decrease over time as the oxygen concentration in the headspace decreases, which will affect cell growth under stress as well as non‐stress conditions. Additionally, the OTR should decrease with increasing liquid volume due to the cylindrical shape of the vials. Therefore, slower growth would be expected due to oxygen transfer limitations. On the other hand, a low culture volume—and hence reduced layer thickness—could have an effect on image analysis by the Growth Profiler software due to reduced contrast affecting Green Values. To find the right balance between these two factors, *P. taiwanensis* GRC2 was cultured in 5 mL glass vials with different volumes of MSM ranging from 400 to 1000 µL, corresponding to filling volumes of 8%–20% (Figure 2C). Based on the obtained results, a culture volume of 600 µL was found to give a good minimum/maximum range of Green Value (ΔGreen Value) (Figure 2C). Culture volumes > 600 µL showed a decrease in maximum Green Value compared to 600 µL when the stationary phase was reached (Figure 2C), indicating oxygen limitation in the system.

Assuming an oxygen content of 21% (v/v) in the headspace and oxygen solubility of 9.1 mg L^−1^ at 20°C in the medium, the total amount of oxygen in the system was calculated to be 38.0 µmol for 600 µL liquid volume when culture preparation is performed at 20°C and 101.3 kPa air pressure. Under these conditions, the oxygen in the system would be sufficient to oxidize 6.3 µmol glucose if biomass formation is not considered. When medium supplemented with 20 mM glucose is used, the total amount in the system calculates to 12.0 µmol. This correlates to about double the amount of glucose that could theoretically be oxidized, indicating that oxygen is most likely the growth‐limiting factor in the system. Applying a biomass yield coefficient *Y_X/S_* = 0.396 ± 0.003 g_CDW_ g_glucose_^−1^ for *P. taiwanensis* GRC2 in MSM supplemented with 20 mM glucose under non‐stress conditions [22], the theoretical oxygen demand would be 43.5 µmol for complete consumption of 20 mM glucose in 600 µL medium when considering biomass formation. This theoretical demand is higher than the calculated amount of oxygen present in the closed system (38.0 µmol), but HPLC analysis of culture supernatant confirmed complete consumption of the carbon source in all tested biological replicates (*n* = 3, data not shown). This indicated that the oxygen supply in the system is sufficient for the complete metabolization of the supplemented carbon source under the chosen non‐stress conditions. Therefore, 600 µL liquid volume was selected for the 5 mL vials, corresponding to a filling volume of 12%. However, under solvent stress conditions—leading to reduced biomass formation and increased respiration rate—the oxygen demand will be higher. In this case, oxygen will likely be the growth limiting factor in the system instead of glucose, depending on the level of solvent exposure. However, this will not affect the early growth phase of cultures, which still allows comparison between different strains.

### 3.2 Comparison of OD_600_ With Green Values

**FIGURE 3:**
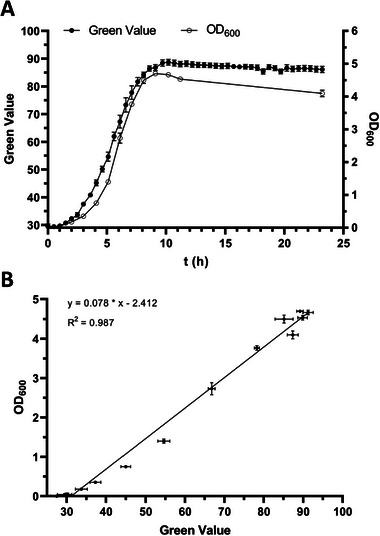
Comparison of Green Value and OD_600_ when using the developed solvent‐tight incubation system in the Growth Profiler. (A) *P. taiwanensis* GRC2 was cultivated in MSM using 5 mL glass vials with 600 µL culture volume. Shown Green Values represent the mean of three biological replicates (*n* = 3) that were monitored over the complete cultivation time. OD_600_ values were obtained from sacrificial sampling. At each sampling point, three biological replicates (*n* = 3) were removed from a pool of parallel‐growing cultures in the Growth Profiler for OD_600_ analysis. (B) Correlation between Green Value and OD_600_ of cultures analyzed by sacrificial sampling. The black line shows linear regression (*y*  =  0.078 × *x* − 2.412). Each data point represents the mean of three biological replicates (*n* = 3), error bars indicate the standard deviation of the mean.

As a final test of the developed cultivation system, Green Values and OD_600_ were monitored in parallel to validate the reliability and accuracy of Green Value–based growth curves when glass vials instead of standard polymer plates were used for Growth Profiler cultivations. Owing to the small culture volume of 600 µL per glass vial, sacrificial sampling was applied for the OD_600_ measurements using a large pool of cultures of *P. taiwanensis* GRC2 grown in parallel. As shown in Figure 3A, although the growth curves are not identical, they follow a similar trend with relatively minor deviations, demonstrating the functionality of the developed system. Linear regression can be used to get an approximation of OD_600_ equivalents from Green Values with a fairly good coefficient of determination (Figure 3B). However, the correlation between the two values strongly depends on the cell morphology, which can be affected by cultivation conditions including solvent‐induced stress. Exposure of bacterial cells to solvents leads to changes in surface structure resulting in a wrinkled appearance [27], which can potentially influence OD_600_ measurements. This means that a reliable calibration curve should always be determined under the chosen experimental conditions, which is challenging, especially in the presence of a second phase of solvents that are known to form emulsions in bacterial cultures [28]. We, therefore, prefer not to convert Green Values to OD_600_ and consider the actually measured Green Values to give a more unbiased representation of cell growth.

### 3.3 Evolving *P. taiwanensis* GRC3 Toward Increased Styrene Tolerance

After we validated that the SIGHT cultivation system is well‐suited for Growth Profiler‐assisted online monitoring of bacterial growth in the presence of solvents, it was applied for ALE of *P. taiwanensis* GRC3 to enhance its tolerance toward styrene. The closed vials prevented evaporation of styrene over time, allowing to maintain constant selection pressure throughout each cultivation cycle. Furthermore, online growth monitoring assisted with culture transfer to fresh medium in a parallelized culture setup when appropriate—despite varying lag phases and growth rates. This massively reduced manual workload and simplified time management.

*P. taiwanensis* GRC3 features inducible solvent tolerance due to the presence of the TtgGHI efflux pump and the regulators TtgVW. Without prior adaptation, the strain is unable to grow in the presence of a second phase of styrene and was selected due to its potential for improvement. The initial styrene concentration for the tolerance ALE was set to 1 mM in the aqueous phase and was stepwise increased by 0.25 mM increments with each cultivation cycle until the solubility limit of about 2.8 mM was reached and a second organic phase was formed. Additionally, three independent cultures of *P. taiwanensis* GRC3 evolved in parallel of which two (cultures I and III) survived all cultivation cycles up to the addition of a second phase of styrene.

**FIGURE 4:**
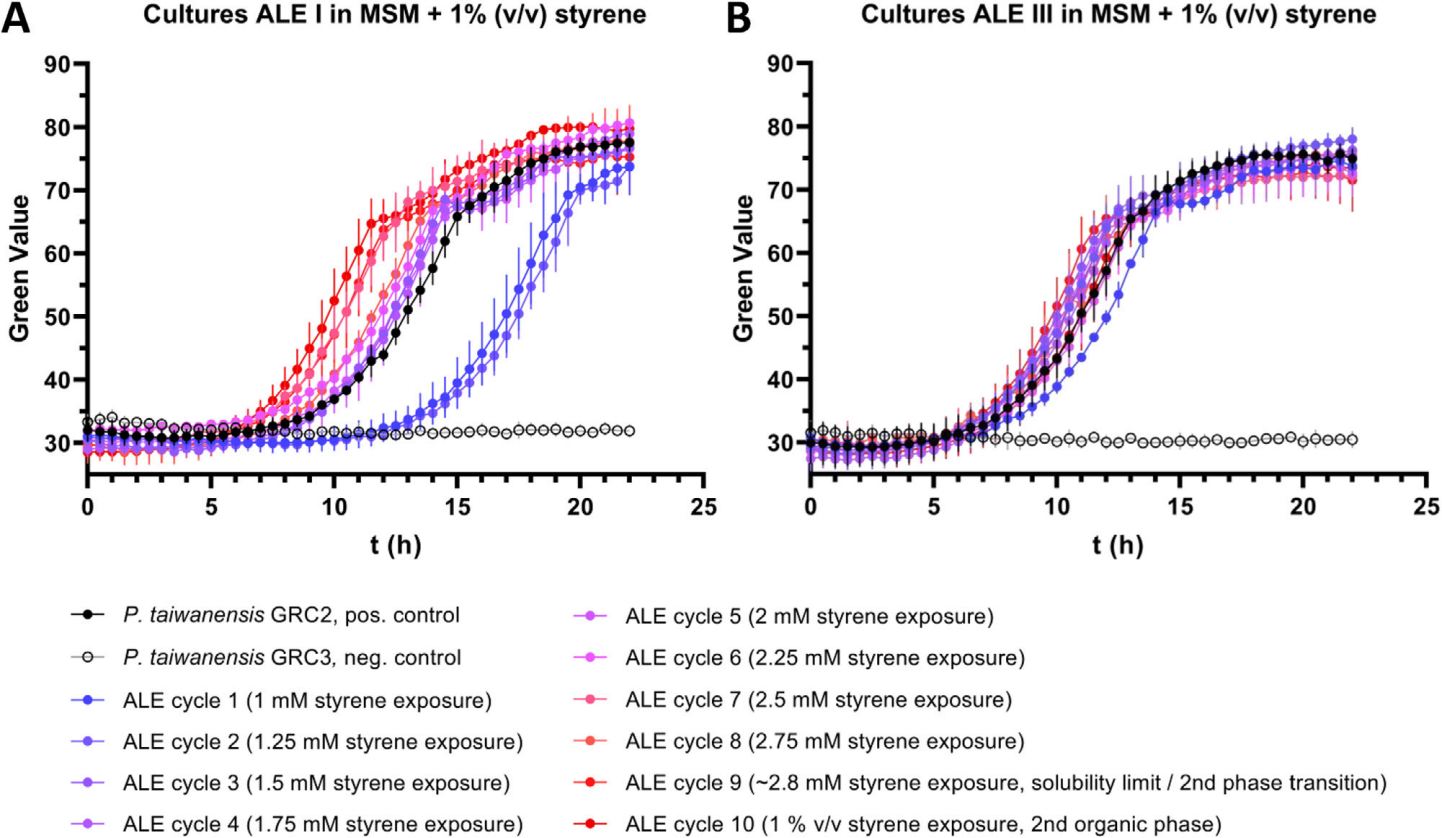
Characterization of heterogenic cryo stocks collected from styrene tolerance ALE of *P. taiwanensis* GRC3. The tested stocks were prepared from cells sampled at the end of each ALE cycle, which were regenerated in LB medium over night to recover from solvent stress. Depicted growth curves show heterogenic cultures originating from these cryo stocks. Cells were cultivated in MSM with addition of 1% (v/v) styrene (second organic phase) without prior adaptation using the SIGHT system in the Growth Profiler. (A) Heterogenic cultures isolated from ALE I. (B) Heterogenic cultures isolated from ALE III. Each data point represents the mean of three biological replicates (*n* = 3), error bars indicate the standard deviation of the mean.

In the first step of characterization, heterogenic cryo stocks collected from each individual ALE cycle of both parallel evolutions were tested for their tolerance toward a second phase of styrene without prior adaptation. The constitutively solvent‐tolerant strain *P. taiwanensis* GRC2 and non‐evolved *P. taiwanensis* GRC3 were used as positive and negative controls, respectively. Cells from all heterogenic stocks were able to grow in MSM with the addition of 1% (v/v) styrene; however, a different pattern of evolution was observed in the two parallel lines (Figure 4). Apparently, exposure to 1 mM of styrene in the first cultivation cycle was sufficient to select for tolerance toward a second phase of styrene without adaptation in both cases. In this context, constitutive solvent tolerance has been previously reported for *P. putida* S12 after surviving shocks with 1% (v/v) toluene [29]. This suggests that the presence of solvents in general—even in low concentrations—might be sufficient to select for increased tolerance phenotypes. Hence, exposure of non‐adapted cells to lethal amounts of solvents such as second‐phase shocks seems not to be the only way to select for high solvent–tolerant phenotypes.

For the stocks isolated from ALE culture I, a stepwise improvement of growth was observed with an increasing number of cultivation cycles. By contrast, stocks isolated from different cycles of ALE culture III showed a more similar growth pattern. The stepwise improvement of culture I could reflect either the clonal enrichment of better‐growing mutants, the accumulation of multiple adaptive mutations within a clone lineage, or both. Based on these results, the focus was set on the analysis of ALE culture I, which was assumed to feature multiple beneficial mutations for potential reverse engineering approaches.

**FIGURE 5:**
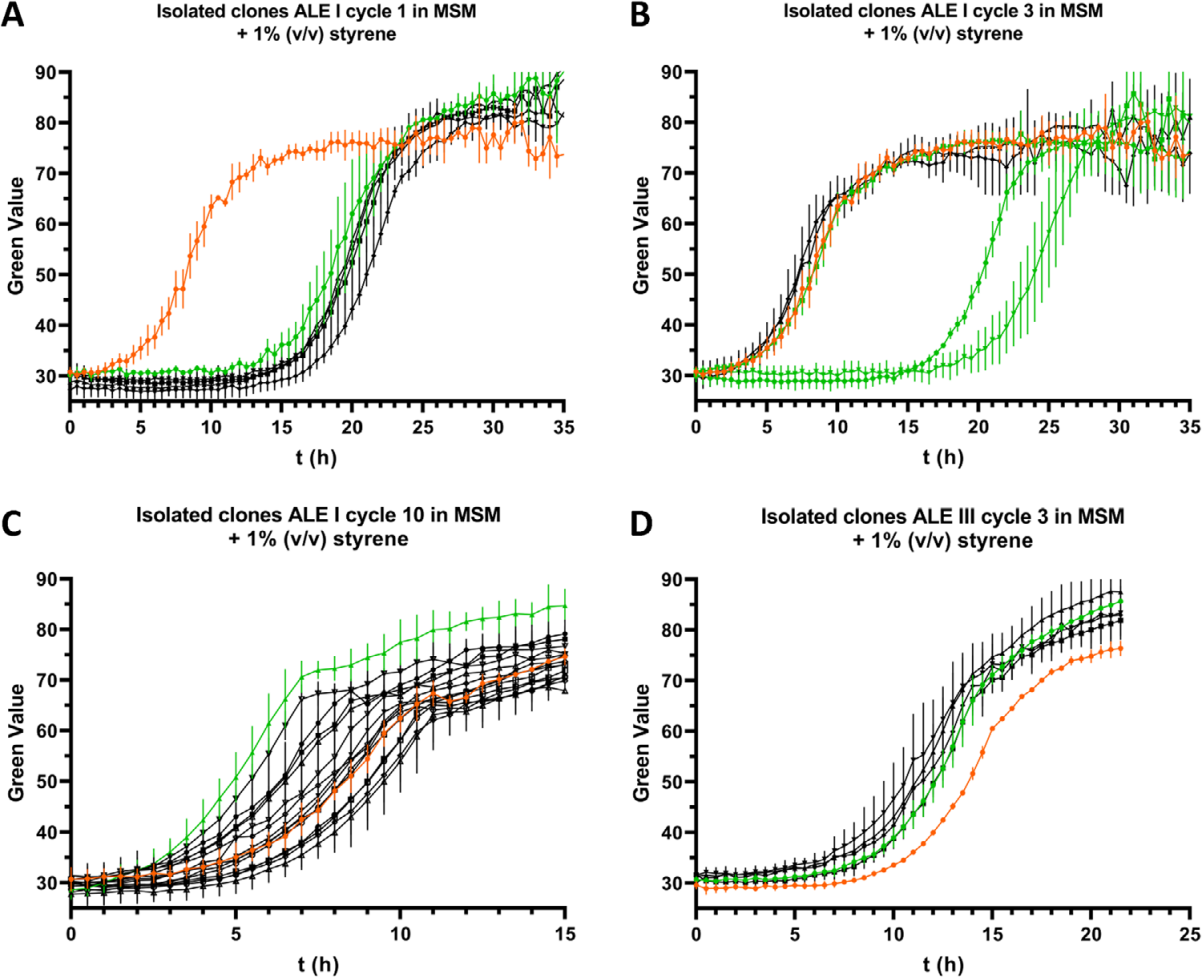
Growth characterization of clones isolated from styrene tolerance ALE of cultures I and III. All clones were cultivated in MSM with the addition of 1% (v/v) styrene (second organic phase) without prior adaptation using the developed solvent‐tight incubation system in combination with the Growth Profiler. Strain *P. taiwanensis* GRC2 (shown in orange) was used as a control to compare growth performance between different clones. The data represents the mean of three biological replicates (*n* = 3), clones selected for genome sequencing are highlighted in green. (A) Five clones isolated from cultivation cycle 1 of culture I with exposure to 1 mM styrene during ALE. (B) Five clones isolated from cultivation cycle 3 of culture I with exposure to 1.5 mM styrene during ALE. (C) A total of 15 clones isolated from cultivation cycle 10 of culture I with exposure to 1% (v/v) styrene (second organic phase) during ALE. (D) Five clones isolated from cultivation cycle 3 of culture III with exposure to 1.5 mM styrene during ALE.

Clones were isolated from the cryo stocks of cultivation cycles 1, 3, and 10 of ALE culture I and characterized regarding their second phase tolerance toward styrene (Figure 5). This approach demonstrates the usefulness of the SIGHT system for analysis of many clones in parallel with a high time resolution, uncovering the emergence of initial adaptive mutants able to grow in the presence of 1% (v/v) styrene in cycle 1 (Figure 5A), a heterogeneous population in cycle 3 (Figure 5B), and eventual enrichment of adaptive mutants that surpass the *P. taiwanensis* GRC2 benchmark strain in terms of growth performance (Figure 5C).

All tested clones from cycle 1 of ALE culture I showed a very similar growth pattern with a prolonged lag phase of about 12 h compared to the *P. taiwanensis* GRC2 control when cultured in MSM + 1% (v/v) styrene (Figure 5A). However, cell growth during the exponential phase was comparable to the control. In the case of clones isolated from cycle 3 of ALE culture I, three out of five tested clones showed an identical growth pattern compared to the *P. taiwanensis* GRC2 control, whereas the other two showed prolonged lag phases (Figure 5B). As the culture sampled from cycle 10 of ALE culture I showed faster growth compared to *P. taiwanensis* GRC2 (Figure 4A), a higher number of clones was isolated and characterized (Figure 5C). Among 15 tested clones, a variation in growth was observed and clone 3 (highlighted in green) was found to be the fastest growing, reaching the stationary phase already after about 7.5 h, whereas some other clones and the control strain *P. taiwanensis* GRC2 needed about 11 h.

For the parallel evolution of ALE culture III (Figure 4B), only cultivation cycle 3 was selected for the isolation and characterization of clones due to the overall lower degree of variation previously observed in the initial screening. The growth of five isolated clones was compared to the control strain *P. taiwanensis* GRC2 (Figure 5D). All of these clones showed a very similar growth pattern compared to each other and had a slightly shorter lag phase than *P. taiwanensis* GRC2.

### 3.4 Genome Sequencing of Clones Obtained From Styrene Tolerance ALE

Out of the characterized clones from parallel styrene tolerance ALE of *P. taiwanensis* GRC3 in cultures I and III, several (highlighted in green within Figure 5) were selected for whole‐genome sequencing to identify the underlying genetic basis of the observed phenotypes. For ALE culture I, clones from all three stages of evolution shown in Figure 5A–C were sequenced, with multiple mutants selected for the heterogeneous cycle 3. An overview of identified mutations found in each sequenced clone is given in Table 2. All identified mutations were additionally verified by Sanger sequencing of the respective locus.

**TABLE 2:** Identified mutations in clones isolated from styrene tolerance ALE of *P. taiwanensis* GRC3.

Evolved in culture	Isolated from cultivation cycle	Styrene exposure during cycle	Clone	Identified mutation(s)	Effect
I	1	1 mM	1	*dnaJ951_952insG*	Frameshift in *dnaJ*
I	3	1.5 mM	1	*dnaJ951_952insG*	Frameshift in *dnaJ*
I	3	1.5 mM	2	*ttgV682_687delGAGC*	Frameshift in *ttgV*
I	3	1.5 mM	4	*dnaJ951_952insG*	Frameshift in *dnaJ*
I	10	1% (v/v)	3	*ttgV682_687delGAGC*, *rpoA769G>C*	Frameshift in *ttgV, rpoA^D257H^*
III	3	1.5 mM	1	*ttgV39_43delins[NC_022738:g.5597919_5623638]*	Transposon integration in *ttgV*

Among the identified genomic mutations (Table 2), the insertion of guanine between nucleotides 951 and 952 in the open reading frame (ORF) of *dnaJ* and the deletion of four base pairs between nucleotides 682 and 687 in *ttgV* resulted in early stop codons and truncation of the respective protein. As a result, the co‐chaperone DnaJ is altered in its amino acid residues from 320 to 344 and additionally truncated by 31 amino acid residues. For TtgV, the repressor of the *ttgGHI* solvent efflux pump operon, the amino acid sequence was altered in positions 231–240 and shortened by 19 amino acid residues. In addition to the truncation, a second mutated variant of *ttgV* was identified, featuring a deletion of nucleotides in positions 39–43 of the ORF and a 25.7 kbp insertion in this position. This insertion resulted from a duplication event of genomic region NC_022738:g.5597919_5623638 containing a putative transposon. At last, a point mutation in *rpoA* encoding the α‐subunit of RNA polymerase (RNAP) was identified causing an amino acid exchange of aspartate 257 to histidine.

### 3.5 Oxygen Limitation Under Solvent Stress Conditions

The aforementioned data demonstrate the usefulness of the SIGHT system for high‐throughput growth monitoring during microbial physiology and evolution experiments. After the characterization of ALE‐derived strains using the SIGHT system, glucose utilization of *P. taiwanensis* GRC2 was investigated under solvent stress conditions, when biomass formation decreased and respiration increased due to increased maintenance demand under solvent stress. It was found that glucose—in the presence of a second phase of styrene—was not depleted when the stationary phase was reached, indicating oxygen limitation. This limitation can be reduced using lower culture volumes. To balance oxygen supply and biomass in the system, *P. taiwanensis* GRC2 was cultivated in MSM with 20 mM glucose and 1% (v/v) styrene using a range of different culture volumes. The culture supernatant was sampled at the end of cultivation when the stationary phase was reached and analyzed via HPLC (Figure S1). Residual glucose was detected under all tested conditions, with 11.6 (±1.1), 7.6 (±2.3), 8.0 (±0.9), and 4.0 (±0.9) mM for culture volumes of 600, 500, 400, and 300 µL, respectively. These results demonstrated that the oxygen demand in the presence of a second phase of styrene was much higher compared to non‐stress conditions. Reduction of cultivation volume—leading to increased oxygen supply due to higher headspace volume in relation to the total biomass in the system—improved glucose utilization but was not sufficient to circumvent oxygen limitation.

## 4 Discussion

### 4.1 Evaluation of the Developed Cultivation System

The SIGHT cultivation system for the Growth Profiler platform presented here further expands the applicability of this device, allowing online growth monitoring of cultures containing solvents or other volatile compounds without risk of evaporation. This system offers several advantages for this purpose compared to cultivation in Boston bottles or closed shake flasks.

The main advantage of the SIGHT system is its high capacity, allowing incubation of up to 240 cultures in parallel with online growth monitoring. This high‐throughput capability was successfully applied for styrene tolerance ALE and characterization of resulting clones as described here, as well as for the screening of solvents for in situ product removal of benzophenones [21].

Compared to other non‐invasive growth monitoring systems such as the CGQ (Scientific Bioprocessing, Inc.) or the BioLector (Beckmann Coulter, Inc.), the SIGHT system is especially designed to prevent the evaporation of volatile compounds. This issue can also be addressed with the CGQ, which is in principle suitable for the monitoring of closed shake flasks; however, the overall capacity is lower. The BioLector, on the other hand, is not suitable for the use of solvents or other volatile compounds due to the use of a gas‐permeable membrane as a sealing of the used microplates. Additionally, only polymer microplates are available for this system, which hinders the use of solvents, as the plate material could be damaged or absorb the solvent.

The major drawback of the use of the SIGHT system is the requirement of the Growth Profiler, which is not a standard laboratory device. However, vial holders conform to a standard deep well plate format, making it potentially suitable for other automated optical monitoring systems, as well as for non‐monitoring shakers with sacrificial sampling for offline analysis without risk of solvent evaporation, as demonstrated in this study.

Another factor is batch‐to‐batch variations in the shape of used glass vials. As these vials are not manufactured for the purpose applied here, different bottom shapes might occur, which could potentially influence optical growth analysis. Although bottom shapes varied markedly in vials tested in this study, we did not observe differences in data quality in relation to these variations, indicating that the online growth monitoring method is relatively robust. However, this could still be different for glass vials from other batches. We, therefore, advise to order glass vials in bulk and validate shape variations for effects on obtained growth curves. Potential batch‐dependent shape variations can also be circumvented by cleaning and re‐using the vials. Based on our experience, durability is high, and we did not notice any issues when re‐using glass vials multiple times. This approach is also cost‐ and resource‐effective, with a relatively minor increase in workload.

### 4.2 Technical Limitations and Other Potential Applications

Along with the benefits of the SIGHT system, there are also some limitations, such as the conversion of Green Values into OD_600_ equivalents. Even though there is a fairly linear correlation between both units when using glass vials, the presence of solvents in the cultivation system can pose difficulties such as emulsification and formation of aggregates consisting of dead cells, which interfere with the image analysis of the Growth Profiler. We, therefore, prefer to show the actual measured Green Values, which give an accurate representation of growth while avoiding potential misinterpretations. Another limitation is the difficulty in accurately monitoring stationary phase cultures in the presence of solvents. In such cases, Green Value data tend to show inconsistent increases over time due to aggregate formation in the presence of solvents, rendering it unreliable and distorting growth curves. This is, however, a general issue and not specific to the system presented here, as emulsification can also affect OD_600_ measurements. Due to the limited availability of oxygen in the headspace, the SIGHT system is also not suitable for high cell–density applications; however, this is also a general issue with closed‐bottle cultures at any scale.

Oxygen availability was found to be growth‐limiting when cells were exposed to a second phase of styrene under the tested conditions. High solvent exposure leads to reduced biomass formation and increased respiration to stabilize the proton gradient required for the activity of the RND‐type solvent efflux pump TtgGHI [17]. Under these conditions, supplemented glucose was not completely consumed, which indicated that oxygen is the limiting factor in the system. This is also supported by calculations regarding the theoretical oxygen concentration in the system, which indicate that under the chosen conditions oxygen demand of the non‐stressed control (43.5 µmol) was higher than the amount present in the system (38.0 µmol). However, all carbon source was completely depleted under these conditions, which might be explained by the flexibility of the electron transport chain of *Pseudomonas*. This allows adaptation to varying oxygen availability and solvent exposure to increase respiration efficiency, as has been reported for *P. putida* [30, 31], which has equivalent terminal oxidases to *P. taiwanensis*.

Nevertheless, oxygen limitation under stress conditions is likely to occur in the closed system and the OTR between gas phase and medium will decrease over time. Reduced maximum Green Values compared to non‐stress conditions are, therefore, not only a result of growth inhibition by styrene but also due to oxygen limitation. Hence, stationary phase data should be interpreted with caution. On the other hand, growth data from the stationary phase is in general not very useful in the presence of a second phase of styrene due to emulsification as mentioned above. For the intended purpose presented here—growth monitoring and strain comparison—limited oxygen supply in the system should not be problematic as it does not affect the early phase of cultivation which is far more relevant for the evaluation of growth performance. Reduction of culture volume down to 300 µL tested in this study improved glucose utilization to some extent; however, this reduction leads to a thinner liquid layer in the vials, which affects image analysis by the Growth Profiler, resulting in a lower Green Value range and quality of growth curves. Alternatively, the amount of supplemented carbon source could be reduced to ensure its complete consumption, or the amount of oxygen in the headspace could be increased by flushing the vials with pure oxygen. Increasing the headspace volume independently from the culture volume by using taller glass vials with equal diameter is not possible, as the dimensions of the Growth Profiler restrict the overall height. The vials used in this study are already at the maximum height that still allows mounting of the SIGHT system to the Growth Profiler. Overall, this means that the boundary conditions of the system such as culture volume and the amount of provided carbon and oxygen need to be considered depending on the intended application, type of medium, and organism used.

In addition to its technical limitations, this system can also be used for direct analysis of the gas phase through GC measurements using headspace injection. This method does not require sample preparation, and the main advantage is that the system remains closed until the measurement, which prevents the evaporation of volatile compounds. However, glass vials used for cultivation need to be compatible with the autosampler of the GC. Therefore, we designed prototype SIGHT racks that fit either 48 standard GC vials of 2 mL total volume or 12 larger headspace injection vials of 11 mL volume. Furthermore, custom templates for the GP960Viewer software are required to enable image analysis, as these racks use non‐standard well layouts. Information and instructions are available in Figures S2–S4 and Table S2 in the Supporting Information to this article. Both prototypes were not characterized in detail in the scope of this study, but have proven to be functional.

Other than the applications tested in this study, there are further potential uses of the SIGHT system. An example would be long‐term cultivations of slow‐growing microorganisms, where the closed system could prevent evaporation and associated reduction of culture volume over time. By using screw caps with appropriate septa, the headspace of the glass vials could also be purged with nitrogen, enabling anaerobic cultivations. Furthermore, the system might also be suitable for gas fermentation, although small headspace volumes would limit the amount of gas that can be supplied to the culture.

### 4.3 High‐Throughput Strain Characterization Supports the Identification of Beneficial Mutations in Evolved Strains

*P. taiwanensis* GRC3 successfully evolved toward increased styrene tolerance in two independent replicates, ALE I and ALE III. For such experiments, identification of adaptive mutations for reverse engineering approaches is the main objective but requires isolation and characterization of promising candidates prior to whole‐genome sequencing. The high‐throughput capability of the SIGHT cultivation system presented here massively reduced the workload of this process as the growth of solvent‐containing cultures can be monitored without manual sampling. Additionally, the detailed growth data in combination with genome sequencing results allowed a better understanding of the adaptation processes during the evolution.

The characterization of heterogenic cultures from each cultivation cycle of the respective ALE culture in the presence of 1% (v/v) styrene revealed different patterns of evolution between both replicates. For all cultures isolated from ALE III, the growth of cells from all cultivation cycles was similar, suggesting that one highly beneficial mutation—presumably inactivation of *ttgV*—occurred during the first cycle, which then dominated throughout the entire experiment. The evolution observed in ALE culture I, on the other hand, suggested the occurrence of mutations with lower fitness improvements in the early stage of the ALE, which were later outperformed by more advantageous ones. This hypothesis is supported by the fact that C‐terminal truncation of the co‐chaperone encoding gene *dnaJ* was only identified in clones isolated from cultivation cycles 1 and 3 but not in combination with *ttgV* inactivation or the *rpoA^D257H^* variant. Furthermore, the presence of identical *ttgV* mutations in *P. taiwanensis* GRC3 ALE I 1.5 mM clone 1 isolated from cultivation cycle 3 and *P. taiwanensis* GRC3 ALE I 1% (v/v) isolated from cultivation cycle 10 indicates that the *rpoA^D257H^* mutation in the latter strain occurred after the *ttgV* inactivation.

### 4.4 Inactivation of *ttgV* Increases Constitutive Styrene Tolerance

Among the genomic mutations obtained from whole‐genome sequencing of strains isolated from the styrene tolerance ALE, a prominent target was the *ttgVW* operon, which was compromised in three out of six sequenced clones. More specifically, the repressor encoded by *ttgV*—responsible for the regulation of the *ttgGHI* solvent efflux pump operon—was either inactivated through a C‐terminal truncation or transposon insertion. The TtgGHI efflux pump belongs to the RND family and represents the most relevant detoxification mechanism for solvents in *P. taiwanensis* VLB120 [22]. Homologs of this efflux pump are also present in other solvent‐tolerant Pseudomonads such as *P. putida* DOT‐T1E [9] and *P. putida* S12, referred to as SrpABC in the latter strain [15]. This efflux pump is expressed at a basal level but is also inducible in the presence of solvents such as styrene or toluene, causing release of the TtgV repressor from its binding site upstream (UP) of the operon [32]. By contrast, the function of TtgW is not yet clear, but it was previously shown by Rojas et al. [32] that *ttgW* deletion does not result in a phenotype when *ttgV* is intact, indicating no regulatory effect on the *ttgGHI* operon. Deletion or inactivation of *ttgV*, on the other hand, leads to a constitutive expression of *ttgGHI*, resulting in high solvent tolerance [19]. Aside from this study, the occurrence of a loss‐of‐function mutation in an efflux pump regulator was previously reported for ALE of pTTS12‐cured *P. putida* S12 toward toluene [33].

### 4.5 C‐Terminal DnaJ Truncation Helps to Cope With Solvent Shocks

In this study, three out of six sequenced clones isolated from the styrene tolerance ALE of culture I showed a mutation, causing the truncation of the co‐chaperone DnaJ. DnaJ belongs to the Hsp40 family and regulates the ATPase activity of its corresponding chaperone DnaK (Hsp70 family) during the back‐folding of damaged proteins, thereby regulating its chaperone activity [34]. Structurally, DnaJ consists of a J domain, which is required for interaction with Hsp70 proteins such as DnaK, a Gly/Phe‐rich region, four cysteine repeats that form two zinc‐binding sites, and a C‐terminal domain (CTD) [35, 36]. Furthermore, DnaJ is capable of binding misfolded proteins, preventing them from aggregating, and directing them to DnaK. For substrate binding, it has been proposed that DnaJ forms ω‐shaped homodimers via the CTD, providing a cavity for interaction with the misfolded protein [37]. Together with the nucleotide exchange factor GrpE, modulating the release of ADP from DnaK after protein folding, these proteins form the DnaK/DnaJ/GrpE chaperone system. This system is part of the heat shock response regulon, which is mainly controlled by the σ^32^ transcription factor in *Escherichia coli* [34, 38]. In Pseudomonads, the regulation of the heat shock response was investigated for strain *P. putida* KT2442 by Ito et al. [39] and described to be similar to that of *E. coli* with regard to DnaK/DnaJ/GrpE. ALE‐derived *P. taiwanensis*. GRC3 strains featuring C‐terminally truncated DnaJ characterized in this study were able to grow in the presence of 1% (v/v) styrene without prior adaptation but only after long lag phases. This suggests that an alteration of this heat shock response system is also beneficial for surviving solvent shocks, but the underlying mechanism remains unknown.

### 4.6 *rpoA^D257H^* Further Increases Styrene Tolerance

In addition to a loss‐of‐function mutation in the solvent efflux pump regulator *ttgV*, an amino acid exchange in the RNAP α‐subunit encoded by *rpoA* was identified for the isolated strain *P. taiwanensis* GRC3 ALE I 1% (v/v) clone 3, leading to substitution of L‐aspartate to L‐histidine in position 257. The RNAP α‐subunit is conserved among bacteria and consists of an N‐terminal (α‐NTD) and C‐terminal (α‐CTD) domain, which are connected by a flexible linker. In terms of function, the α‐subunit is involved in the assembly of the RNAP complex, DNA binding, and interaction with transcriptional activators [40, 41]. The exchanged amino acid residue in position 257 is located within the α‐CTD, which contains a non‐standard helix (NSH) followed by four α‐helices (α1, α2, α3, and α4) [42]. Therefore, the NSH motif (ILLRPV) is directly adjacent to amino acid residue 257, which could potentially affect its structure.

Based on the known interaction of the α‐CTD with UP elements and transcription activators [40], the identified mutation could change the expression pattern of a variety of genes, which would explain the increased solvent‐tolerant phenotype. Additionally, mutations in RNAP subunits have been previously reported in the context of solvent tolerance. Kusumawardhani et al. [33] identified a mutation in RPPX_06985 encoding the RNAP β′ subunit in *P. putida* S12, which increased tolerance toward toluene. For *E. coli*, the truncation of the α‐CTD of RNAP α‐subunit encoded by *rpoA* was reported to result in higher butanol tolerance [43].

## 5 Conclusion

In this study we designed, tested, and validated the SIGHT cultivation system consisting of commercially available glass vials and a 3D‐printed vial holder. This system is compatible with the Growth Profiler (EnzyScreen), allowing small‐scale cultivations with online growth monitoring in the presence of solvents—or volatile compounds in general, without risk of evaporation. Aside from the use of this cultivation system presented here, the standardized microtiter plate footprint makes it also compatible with other devices and robotic platforms, further extending its range of applications. Compared to conventional systems requiring manual sampling for growth monitoring, the automated image analysis–based monitoring of the Growth Profiler massively reduces manual workload and increases temporal flexibility, which is often an issue with solvent tolerance experiments. The system was successfully applied for ALE of *P. taiwanensis* GRC3 toward increased styrene tolerance and proved to be very useful for the subsequent characterization of isolated strains due to its high throughput capability. The genome sequencing of selected ALE‐derived clones with increased styrene tolerance revealed not only loss‐of‐function mutations in *ttgV* (already reported in the literature) but also novel mutations in *dnaJ* and *rpoA*. These might be promising targets for future rational engineering of solvent tolerance, but the genes proved intractable to genetic engineering using several established systems. However, our findings provide insights into the genetic adaptation of *P. taiwanensis* during prolonged and increasing exposure to styrene, as well as growth dynamics during and after evolution enabled through the SIGHT system.

## Conflicts of Interest

The authors declare no conflicts of interest.

## Supporting information



Supporting Information

## Data Availability

The NGS data for this study have been deposited in the SRA at NCBI under accession number: PRJNA1104788. STL files for SIGHT racks presented in this study are available at https://www.thingiverse.com/microbial_catalysis/designs.
