# Supplementary material for: SIGHT—A System for Solvent‐Tight Incubation and Growth Monitoring in High Throughput
Source: Eng Life Sci. 2024 Sep 11;25(2):e202400037. doi: 10.1002/elsc.202400037 (PMC11842283; doi:10.1002/elsc.202400037)
Supplement: Supplementary file 1 — Supporting Information [file ELSC-25-e202400037-s001.pdf]

Supplementary material to the article

“SIGHT - A system for solvent-tight incubation and growth monitoring in high throughput”

Jakob Rönitz, Felix Herrmann, Benedikt Wynands, Tino Polen and Nick Wierckx

Institute of Bio- and Geosciences IBG-1: Biotechnology, Forschungszentrum Jülich, 52428 Jülich, Germany

Correspondence: Prof. Dr. Nick Wierckx, E-mail: n.wierckx@fz-juelich.de, Institute of Bio- and Geosciences IBG-1: Biotechnology, Forschungszentrum Jülich, Wilhelm-Johnen-Straße, 52428 Jülich, Germany

Table of contents

Table S1	Primers used in this study.	page 2
Figure S1	Residual glucose in culture supernatant of *P. taiwanensis* GRC2.	page 2
Figure S2	48- and 12-vial SIGHT racks	page 3
Figure S3	Growth curves of *P. taiwanensis* GRC3 in 48- and 12-vial SIGHT racks	page 3
Table S2	Glass vial parts used for 48- and 12-vial SIGHT racks	page 4
Figure S4	Instructions for generating custom templates in GP960Viewer	page 5

Table S1: Primers used in this study.

Primer name	Sequence 5'→3'	Description
JR007	CTATTTCGTCCAGCGTAGCGTC	Amplification and Sanger sequencing of *ttgV*
JR038	GGCTATTGCGCAGTTGGTTG	Amplification and Sanger sequencing of *ttgV*
JR008	ATGAGCGTAAGTACGGCTTTACC	Amplification and Sanger sequencing of transposon in *ttgV* (left flank)
JR079	CGTTATCAGCATCGACGGTATCG	Amplification and Sanger sequencing of transposon in *ttgV* (left flank)
JR057	GACTCCAATGCCTGTTGCAGG	Amplification and Sanger sequencing of transposon in *ttgV* (right flank)
JR080	GGTGTAGATGAACGGCTTGTAGTC	Amplification and Sanger sequencing of transposon in *ttgV* (right flank)
JR075	CGTGGTCATCAATGTGCGTGAG	Amplification and Sanger sequencing of *dnaJ*
JR076	CACTGCAATACGTCGCATACGTC	Amplification and Sanger sequencing of *dnaJ*
JR077	CGAGAGACAGACCACGAGAG	Amplification and Sanger sequencing of *rpoA*
JR078	CTCTCGTCAAACTGACGAAGACG	Amplification and Sanger sequencing of *rpoA*

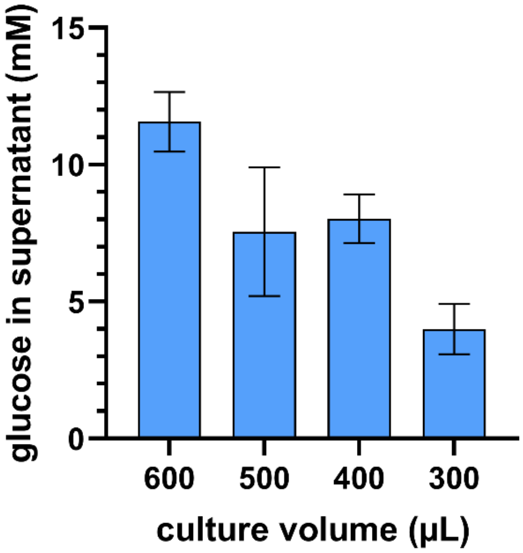

Figure S1: Residual glucose in culture supernatant of *P. taiwanensis* GRC2. Solvent stress was induced by addition of 1% (v/v) styrene. MSM with 20 mM glucose was used and cultures were adjusted to initial OD_{600} of 0.05. Supernatant samples were collected at the end of cultivation after reaching the stationary phase. For each tested culture volume three biological replicates (n = 3) were analysed. Error bars indicate standard deviation of the mean.

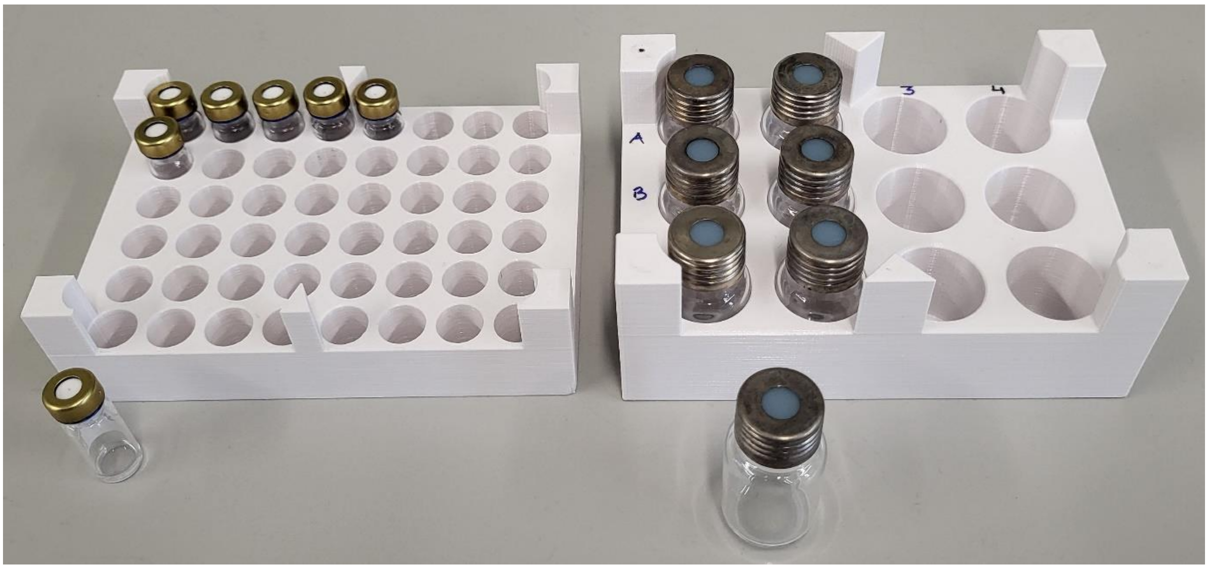

Figure S2: 48- and 12-vial SIGHT racks. The magnetic caps allow the use of autosamplers, enabling analysis of the culture via GC headspace injection without sample preparation. STL files for both racks are available at https://www.thingiverse.com/microbial_catalysis/designs.

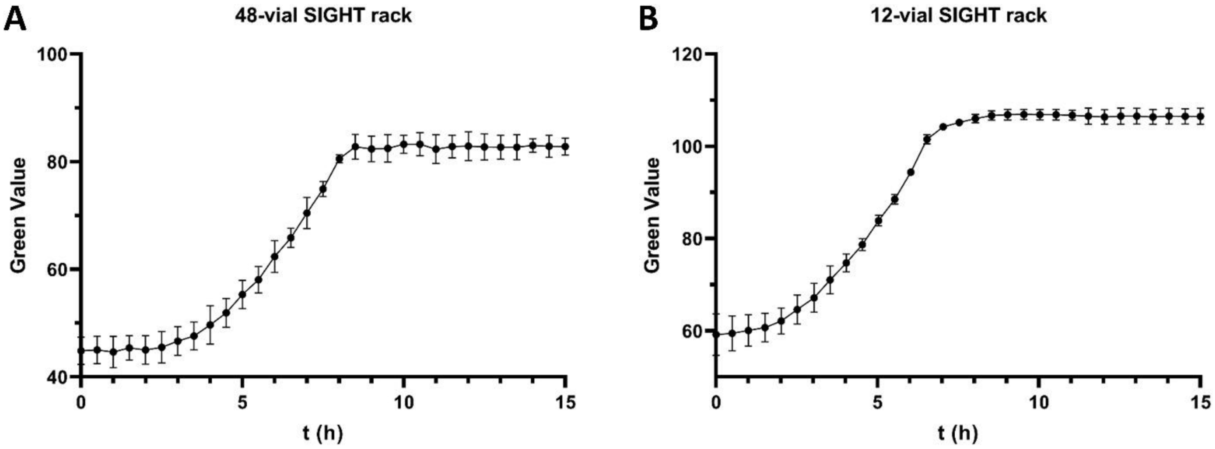

Figure S3: Growth of *P. taiwanensis* GRC3 in different size glass vials for the SIGHT system. **A** Growth in closed 2 mL vials with 200 µL culture volume using a 48-vial SIGHT rack. **B** Growth in closed 11 mL vials with 1 mL culture volume using a 12-vial SIGHT rack. MSM supplemented with 20 mM glucose was used for all cultivations and initial OD_{600} was adjusted to 0.05. Data shown in A and B represents four (n = 4) and three (n = 3) biological replicates, respectively. Error bars indicate standard deviation of the mean.

Table S2: Glass vials and screw caps used for 48- and 12-vial SIGHT racks. The total volume was determined experimentally as described in Material and Methods in the main article.

SIGHT rack capacity	Total vial volume (mL)	Glass vial parts	Supplier
48 vials	1.945 ± 0.009 (n = 6)	PAL System Vial 2CV, 1.5ml Clear Glass with Label (Art. No. C-VIAL-1.5-ND9-CG-100)	BGB Analytik AG, Switzerland
		ND9 Magnetic Short Thread Screw Caps (gold) with Septa Silicone/PTFE (Art. No. 090301-M)	BGB Analytik AG, Switzerland
12 vials	11.092 ± 0.093 (n = 7)	HS-Thread Vial G 10, clear (Art. No. 301236-01)	CS Chromatographie Service GmbH, Germany
		magn. HS-Screw Cap G 18/SIL, clear-white (Art. No. 301346-01)	CS Chromatographie Service GmbH, Germany

Step 1: select MTP Geometry in Settings tab of GP960Viewer.
Step 2: click Add New.
Step 3: add new templates with indicated settings. Note: a 4x4 layout is required for the 12-well plate, otherwise images are not analysed correctly. The resulting additional row with wells D1-D4 can be ignored.
Step 4: set Deviation from Center in Y Dir. to 13 mm (for 12-well plate only).

1. 2. 3.

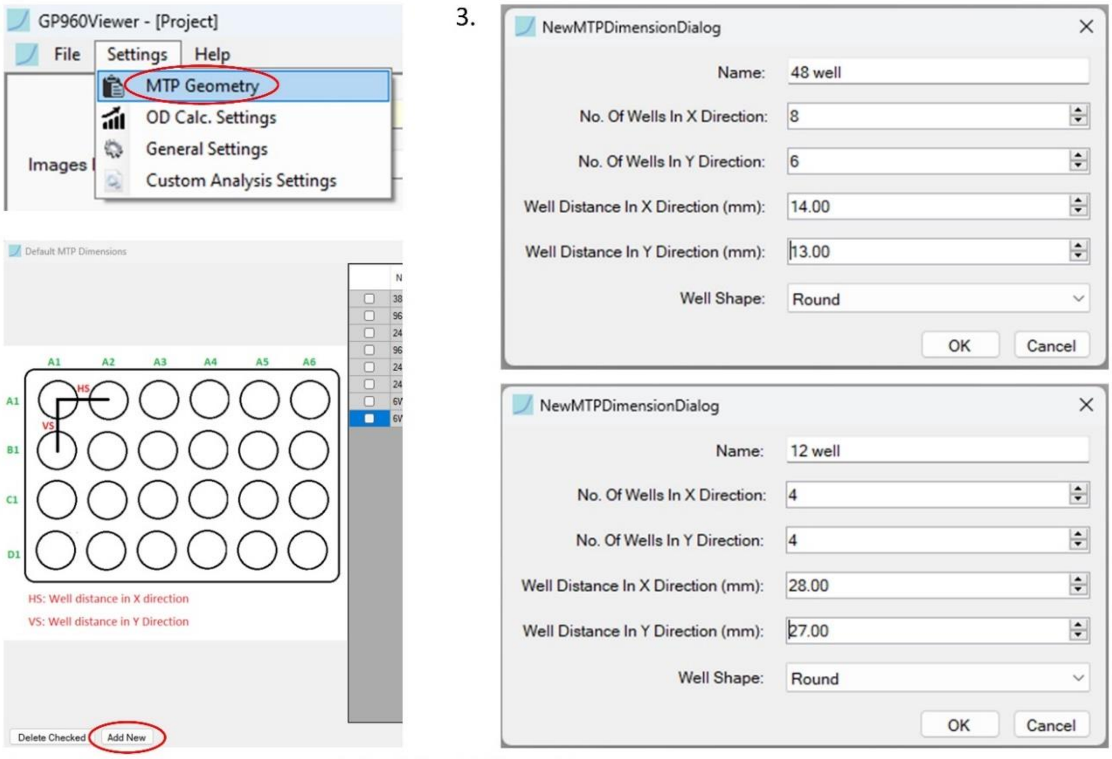

4.

Image Analysis Settings: Message: Analysis Completed Successfully

MTP No.	Copy Default MTP Dimensions	No. of Wells In X Dir.	No. of Wells In Y Dir.	Well Distance In X Dir. (mm)	Well Distance In Y Dir. (mm)	Well Shape	Spot Shape	Size Spot In X Direction (mm)	Size Spot In Y Direction (mm)	Deviation From Center in X Dir. (mm)	Deviation From Center in Y Dir. (mm)	Deviation From Perfect Parallelne (deg.)
1	48 well	8	6	14	13	Round	Rectangle	3	3	0	0	0
2	12 well	4	4	28	27	Round	Rectangle	3	3	0	13	0

Figure S4: Instructions for generating custom well plate layouts in GP960Viewer in order to enable image analysis when using 48- and 12-vial SIGHT racks in the Growth Profiler.
